# Psychosocial risks in the practice of healthcare professionals: from the culture of stoicism to occupational suicide.

**DOI:** 10.1192/j.eurpsy.2024.443

**Published:** 2024-08-27

**Authors:** V. S. D. Melo, I. A. Silva, A. F. Silva, A. Gouveia, C. A. Rodrigues

**Affiliations:** ^1^Psychiatry, Centro Hospitalar do Médio Tejo, Tomar; ^2^Psychiatry, ULSNA, Portalegre; ^3^Psychiatry, ULSBA, Beja, Portugal

## Abstract

**Introduction:**

It is well known that healthcare professionals, in a somewhat generalized manner, work in stressful contexts that embrace emotional overload, highly hierarchical environments, and not always sensitive to the vulnerabilities that arise. Chronic professional stress in institutions, associated with the perception of low control and emotional exhaustion, acts as a trigger for eminently deleterious consequences, significantly affecting the most dedicated and perfectionist professionals.

**Objectives:**

This work aims, through a non-systematic literature review, to analyze the psychosocial risks associated with the practice of healthcare professionals, as well as the mitigation strategies whose practical implementation may depend on and maintenance of a positive and protective occupational environment.

**Methods:**

For the purpose of literature review, a search was conducted on search engines such as Google Scholar, Research Gate, and PubMed, with no date limitations, using the following terms (or combinations): “occupational psychiatry”; “psychosocial risks AND healthcare professionals”; “mitigation strategies”; “occupational risk management.”

**Results:**

Healthcare sector professionals are the ones reporting exposure to higher levels of workload intensity, including parameters related to work speed (under time pressure), combined with prominent emotional demands and psychological suffering. In addition to the most commonly analyzed occupational stressors (workload, job fatigue, particularly draining emotional interactions, marked cognitive demands, complex decision-making, conflicts of a deontological nature), other relevant contextual factors emerge. Among these, predisposing personality traits (such as neuroticism), a sense of personal sacrifice with neglect of self-care, vicarious trauma, which is intimately related to compassion fatigue in the face of frequent and prolonged exposure to traumatic experiences (of various kinds) of the patients they accompany, and occupational violence, which can manifest as verbal or behavioral threats, mobbing, physical harm, and/or sexual abuse based on a tendentially gender-based and deeply hierarchical structure.

**Conclusions:**

Undeniable consequences such as job dissatisfaction, psychological distress, the development of anxiety, depression, burnout, and post-traumatic stress disorder translate into a loss of capacity to perform occupational functions, with a higher risk of medical/clinical errors, conferring risks that should never be neglected to the safety of the users of healthcare institutions. On the darker side of this panorama, and in the face of chronic depletion, occupational suicide emerges. It is therefore urgent to requalify the work environment, aiming at creating and maintaining a positive occupational environment, or alternatively, a preventive approach to the risk of mental health problems originating or exacerbated in the workplace.

**Disclosure of Interest:**

None Declared

